# Supplementary material for: Pyruvate Dehydrogenase Kinase 1 inhibition mediated oxidative phosphorylation enhancement in cartilage promotes osteoarthritis progression
Source: BMC Musculoskelet Disord. 2023 Jul 20;24:597. doi: 10.1186/s12891-023-06585-6 (PMC10357736; doi:10.1186/s12891-023-06585-6)
Supplement: Supplementary file 1 — Additional file 1. [file 12891_2023_6585_MOESM1_ESM.zip › BP.Gsea.1653623667859/GOBP_GLUTATHIONE_METABOLIC_PROCESS.html]

Details for gene set GOBP\_GLUTATHIONE\_METABOLIC\_PROCESS[GSEA]

|  || Dataset | 55457\_collapsed\_to\_symbols.cls.cls #OA\_versus\_normal.cls.cls #OA\_versus\_normal\_repos |
| Phenotype | cls.cls#OA\_versus\_normal\_repos |
| Upregulated in class | OA |
| GeneSet | GOBP\_GLUTATHIONE\_METABOLIC\_PROCESS |
| Enrichment Score (ES) | 0.3932163 |
| Normalized Enrichment Score (NES) | 1.6337317 |
| Nominal p-value | 0.010230179 |
| FDR q-value | 0.22662401 |
| FWER p-Value | 1.0 |
Table: GSEA Results Summary

  

Fig 1: Enrichment plot: GOBP\_GLUTATHIONE\_METABOLIC\_PROCESS      
 Profile of the Running ES Score & Positions of GeneSet Members on the Rank Ordered List

  

| SYMBOL | TITLE | RANK IN GENE LIST | RANK METRIC SCORE | RUNNING ES | CORE ENRICHMENT || 1 | GSTA4 | glutathione S-transferase alpha 4 [Source:HGNC Symbol;Acc:HGNC:4629] | 216 | 0.808 | 0.0332 | Yes |
| 2 | GSTK1 | glutathione S-transferase kappa 1 [Source:HGNC Symbol;Acc:HGNC:16906] | 490 | 0.662 | 0.0529 | Yes |
| 3 | GSS | glutathione synthetase [Source:HGNC Symbol;Acc:HGNC:4624] | 502 | 0.658 | 0.0929 | Yes |
| 4 | ETHE1 | ETHE1 persulfide dioxygenase [Source:HGNC Symbol;Acc:HGNC:23287] | 572 | 0.632 | 0.1266 | Yes |
| 5 | CLIC3 | chloride intracellular channel 3 [Source:HGNC Symbol;Acc:HGNC:2064] | 582 | 0.629 | 0.1649 | Yes |
| 6 | HPGDS | hematopoietic prostaglandin D synthase [Source:HGNC Symbol;Acc:HGNC:17890] | 605 | 0.622 | 0.2017 | Yes |
| 7 | MMACHC | metabolism of cobalamin associated C [Source:HGNC Symbol;Acc:HGNC:24525] | 642 | 0.608 | 0.2365 | Yes |
| 8 | GSTZ1 | glutathione S-transferase zeta 1 [Source:HGNC Symbol;Acc:HGNC:4643] | 974 | 0.523 | 0.2431 | Yes |
| 9 | GSTM3 | glutathione S-transferase mu 3 [Source:HGNC Symbol;Acc:HGNC:4635] | 1025 | 0.512 | 0.2710 | Yes |
| 10 | GSTT1 | glutathione S-transferase theta 1 [Source:HGNC Symbol;Acc:HGNC:4641] | 1129 | 0.491 | 0.2933 | Yes |
| 11 | CLIC2 | chloride intracellular channel 2 [Source:HGNC Symbol;Acc:HGNC:2063] | 1214 | 0.473 | 0.3161 | Yes |
| 12 | GSTP1 | glutathione S-transferase pi 1 [Source:HGNC Symbol;Acc:HGNC:4638] | 1260 | 0.465 | 0.3414 | Yes |
| 13 | HAGH | hydroxyacylglutathione hydrolase [Source:HGNC Symbol;Acc:HGNC:4805] | 1395 | 0.443 | 0.3584 | Yes |
| 14 | PARK7 | Parkinsonism associated deglycase [Source:HGNC Symbol;Acc:HGNC:16369] | 1406 | 0.442 | 0.3850 | Yes |
| 15 | NFE2L2 | "nuclear factor, erythroid 2 like 2 [Source:HGNC Symbol;Acc:HGNC:7782]" | 2034 | 0.357 | 0.3583 | Yes |
| 16 | GLO1 | glyoxalase I [Source:HGNC Symbol;Acc:HGNC:4323] | 2141 | 0.345 | 0.3715 | Yes |
| 17 | SOD1 | superoxide dismutase 1 [Source:HGNC Symbol;Acc:HGNC:11179] | 2413 | 0.313 | 0.3697 | Yes |
| 18 | ARL6IP5 | ADP ribosylation factor like GTPase 6 interacting protein 5 [Source:HGNC Symbol;Acc:HGNC:16937] | 2545 | 0.298 | 0.3780 | Yes |
| 19 | MGST2 | microsomal glutathione S-transferase 2 [Source:HGNC Symbol;Acc:HGNC:7063] | 2583 | 0.292 | 0.3932 | Yes |
| 20 | GSTM5 | glutathione S-transferase mu 5 [Source:HGNC Symbol;Acc:HGNC:4637] | 3123 | 0.238 | 0.3660 | No |
| 21 | GSTM4 | glutathione S-transferase mu 4 [Source:HGNC Symbol;Acc:HGNC:4636] | 3144 | 0.235 | 0.3790 | No |
| 22 | CTNS | "cystinosin, lysosomal cystine transporter [Source:HGNC Symbol;Acc:HGNC:2518]" | 3337 | 0.218 | 0.3775 | No |
| 23 | GSTA3 | glutathione S-transferase alpha 3 [Source:HGNC Symbol;Acc:HGNC:4628] | 3807 | 0.176 | 0.3519 | No |
| 24 | GSR | glutathione-disulfide reductase [Source:HGNC Symbol;Acc:HGNC:4623] | 4597 | 0.105 | 0.2970 | No |
| 25 | CLIC1 | chloride intracellular channel 1 [Source:HGNC Symbol;Acc:HGNC:2062] | 4704 | 0.095 | 0.2947 | No |
| 26 | GGT1 | gamma-glutamyltransferase 1 [Source:HGNC Symbol;Acc:HGNC:4250] | 5177 | 0.058 | 0.2615 | No |
| 27 | OPLAH | "5-oxoprolinase, ATP-hydrolysing [Source:HGNC Symbol;Acc:HGNC:8149]" | 5339 | 0.045 | 0.2518 | No |
| 28 | GSTM1 | glutathione S-transferase mu 1 [Source:HGNC Symbol;Acc:HGNC:4632] | 5848 | 0.005 | 0.2125 | No |
| 29 | GLRX2 | glutaredoxin 2 [Source:HGNC Symbol;Acc:HGNC:16065] | 6116 | -0.014 | 0.1926 | No |
| 30 | PTGES2 | prostaglandin E synthase 2 [Source:HGNC Symbol;Acc:HGNC:17822] | 6269 | -0.025 | 0.1823 | No |
| 31 | GSTO1 | glutathione S-transferase omega 1 [Source:HGNC Symbol;Acc:HGNC:13312] | 6275 | -0.025 | 0.1835 | No |
| 32 | SLC7A11 | solute carrier family 7 member 11 [Source:HGNC Symbol;Acc:HGNC:11059] | 6470 | -0.040 | 0.1709 | No |
| 33 | GGTLC1 | gamma-glutamyltransferase light chain 1 [Source:HGNC Symbol;Acc:HGNC:16437] | 6916 | -0.073 | 0.1408 | No |
| 34 | SLC1A2 | solute carrier family 1 member 2 [Source:HGNC Symbol;Acc:HGNC:10940] | 7026 | -0.082 | 0.1374 | No |
| 35 | G6PD | glucose-6-phosphate dehydrogenase [Source:HGNC Symbol;Acc:HGNC:4057] | 7082 | -0.086 | 0.1384 | No |
| 36 | IDH1 | isocitrate dehydrogenase (NADP(+)) 1 [Source:HGNC Symbol;Acc:HGNC:5382] | 7109 | -0.089 | 0.1419 | No |
| 37 | GPX4 | glutathione peroxidase 4 [Source:HGNC Symbol;Acc:HGNC:4556] | 7115 | -0.089 | 0.1471 | No |
| 38 | GDAP1 | ganglioside induced differentiation associated protein 1 [Source:HGNC Symbol;Acc:HGNC:15968] | 7172 | -0.093 | 0.1485 | No |
| 39 | GSTT2 | glutathione S-transferase theta 2 (gene/pseudogene) [Source:HGNC Symbol;Acc:HGNC:4642] | 7666 | -0.132 | 0.1183 | No |
| 40 | SLC1A1 | solute carrier family 1 member 1 [Source:HGNC Symbol;Acc:HGNC:10939] | 8157 | -0.167 | 0.0905 | No |
| 41 | GCLC | glutamate-cysteine ligase catalytic subunit [Source:HGNC Symbol;Acc:HGNC:4311] | 8534 | -0.196 | 0.0734 | No |
| 42 | GSTT4 | glutathione S-transferase theta 4 [Source:HGNC Symbol;Acc:HGNC:26930] | 8923 | -0.226 | 0.0572 | No |
| 43 | DPEP1 | dipeptidase 1 [Source:HGNC Symbol;Acc:HGNC:3002] | 9353 | -0.260 | 0.0399 | No |
| 44 | GGT5 | gamma-glutamyltransferase 5 [Source:HGNC Symbol;Acc:HGNC:4260] | 9361 | -0.261 | 0.0555 | No |
| 45 | CLIC5 | chloride intracellular channel 5 [Source:HGNC Symbol;Acc:HGNC:13517] | 9382 | -0.262 | 0.0702 | No |
| 46 | CHAC1 | ChaC glutathione specific gamma-glutamylcyclotransferase 1 [Source:HGNC Symbol;Acc:HGNC:28680] | 9853 | -0.307 | 0.0526 | No |
| 47 | NAT8 | N-acetyltransferase 8 (putative) [Source:HGNC Symbol;Acc:HGNC:18069] | 9879 | -0.310 | 0.0698 | No |
| 48 | GCLM | glutamate-cysteine ligase modifier subunit [Source:HGNC Symbol;Acc:HGNC:4312] | 10439 | -0.366 | 0.0489 | No |
| 49 | GDAP1L1 | ganglioside induced differentiation associated protein 1 like 1 [Source:HGNC Symbol;Acc:HGNC:4213] | 10607 | -0.384 | 0.0597 | No |
| 50 | GSTA1 | glutathione S-transferase alpha 1 [Source:HGNC Symbol;Acc:HGNC:4626] | 11608 | -0.517 | 0.0139 | No |
| 51 | EEF1G | eukaryotic translation elongation factor 1 gamma [Source:HGNC Symbol;Acc:HGNC:3213] | 11767 | -0.544 | 0.0353 | No |
| 52 | SOD2 | superoxide dismutase 2 [Source:HGNC Symbol;Acc:HGNC:11180] | 12685 | -0.853 | 0.0167 | No |
Table: GSEA details [plain text format]

  

Fig 2: GOBP\_GLUTATHIONE\_METABOLIC\_PROCESS      
 Blue-Pink O' Gram in the Space of the Analyzed GeneSet

  

Fig 3: GOBP\_GLUTATHIONE\_METABOLIC\_PROCESS: Random ES distribution      
 Gene set null distribution of ES for **GOBP\_GLUTATHIONE\_METABOLIC\_PROCESS**
